# Design and rationale for an empirical investigation of the resource use and costs of investigator-initiated randomized trials in Switzerland, the UK, and Germany

**DOI:** 10.1186/s13063-024-08505-y

**Published:** 2024-10-07

**Authors:** Alexandra Griessbach, Benjamin Speich, Gilles Dutilh, Shaun Treweek, Matthias Schwenkglenks, Matthias Briel, on behalf of the MAking Randomized Trials Affordable (MARTA) Group

**Affiliations:** 1 CLEAR Methods Center, Division of Clinical Epidemiology, Department Clinical Research, University Hospital Basel and University of Basel, Basel, Switzerland; 2 Department Clinical Research, University Hospital Basel and University of Basel, Basel, Switzerland; 3 Health Services Research Unit, University of Aberdeen, Aberdeen, UK; 4 Institute of Pharmaceutical Medicine (ECPM), University of Basel, Basel, Switzerland; 5 Health Economics Facility, Department of Public Health, University of Basel, Basel, Switzerland; 6 Department of Health Research Methodology, Evidence, and Impact, McMaster University, Hamilton, ON Canada

**Keywords:** RCTs, Costs of clinical trials, Resource use, Costs, Planning, Conduct, Finalization

## Abstract

**Background:**

Conducting high-quality randomized clinical trials (RCTs) is challenging, time consuming, and resource intense. Academic investigators usually depend on scarce financial resources; however, current literature lacks systematically collected empirical data on the detailed resource use and costs of investigator-initiated RCTs.

**Methods:**

The aim of this study is to generate a database of detailed empirical resource use and cost data from 100 investigator-initiated RCTs in Switzerland, Germany, and the UK. Investigators enter their empirical costs data into an online data collection form, which is followed by a short interview and a detailed cost report. We plan to investigate cost patterns and cost drivers and examine planned versus actual RCT costs as well as explore different strata of costs across the planning, conduct, and finalization phases, in drug and non-drug trials, and across medical fields and countries.

**Discussion:**

This study will add detailed empirical data to the limited research on investigator-initiated RCT costs currently available. A study limitation will be that cost data will be retrospective and self-reported, which might be inaccurate depending on how costs were recorded.

**Trial registration:**

Open Science Framework (OSF) https://doi.org/10.17605/OSF.IO/QY2GU. Registered on June 4, 2021.

**Supplementary Information:**

The online version contains supplementary material available at 10.1186/s13063-024-08505-y.

## Background

Randomized clinical trials (RCTs) are the cornerstone of reliable evaluation of interventions in health care, with results that often impact clinical guidelines and evidence-based practice ultimately leading to better health care for patients [1–3]. However, conducting high-quality RCTs is challenging, resource intense, and associated with high costs [4, 5]. Tens of billions of dollars of public and private money are invested in RCTs every year, estimated to reach $68.9 billion a year by 2025 [6]. Moreover, RCT costs are rising. Over the last decade, a number of new initiatives and regulations were implemented to increase participant protection and to improve research quality [2, 7–10]. As a consequence, the complexity and administrative burden of RCTs have increased overall costs [11–15]. Furthermore, the implementation of medical advances, while highly desirable, has limited the incremental impact of new therapies, resulting in smaller beneficial effects of interventions, which requires larger RCT sample sizes [16, 17]. All these developments have resulted in escalating RCT costs, thus exasperating the problem of scarce resources for academic clinical research.

One consequence of these cost pressures is that the number of independent investigator-initiated RCTs has remained constrained over the last two decades [2, 18, 19] and a substantial proportion of these trials are prematurely discontinued due to recruitment and organizational problems [19], which risks many clinical research questions remaining unaddressed. Therefore, efforts to make investigator-initiated RCTs more cost-effective and sufficiently funded are urgently needed.

In spite of this, the literature on how much RCTs actually cost remains sparse [20–22]. It was only in the last few years that the first systematic investigations addressing RCT costs have been published [20, 21, 23]. Sertkaya et al. analyzed aggregated cost data of clinical trials sponsored by the pharmaceutical industry in the USA between 2004 and 2012 and reported average costs of phase III trials ranging from US$ 11.5 million (dermatology) to US$ 52.9 million (pain and anesthesia) and of phase II trials ranging from US$ 7.0 million (cardiovascular) to US$ 19.6 million (hematology) [23]. Apart from trial phase, the authors identified therapeutic area as well as the number and types of clinical procedures involved as key drivers of direct costs in pharmaceutical RCTs. Another study collected cost data directly from seven major biopharma companies including 726 interventional studies conducted from 2010 to 2015 using a standardized approach to assign clinical development spending to individual trials [21]. The median trial costs from protocol approval to the final report proved to be US$ 21.4 million for phase III trials and US$ 8.6 million for phase II trials with huge variability around these averages (e.g., 25th and 75th percentile for phase III trials of US$ 9 million and US$ 44 million, respectively). The authors derived a multivariable model including number of trial sites, therapeutic area, and treatment duration as main independent variables that explained 60–90% of the variance of overall trial costs in phase II and phase III trials. Finally, Raftery et al. reported the analysis of metadata on 125 published RCTs funded by the National Institute for Health Research Health Technology Assessment programme (UK) between 1995 and 2005, including cost data [20]. The authors calculated mean total trial costs ranging between £0.5 million and £2.4 million. They further showed that the actual costs exceeded the planned costs in 78% of projects, with 57% of projects exceeding by 10% or more and 12% exceeding by 50% or more. In multivariable regression analyses, they identified a greater number of trial sites and longer trial duration as the independent variables associated with higher trial costs, explaining 40% of the variance in their model.

None of these three studies provided a detailed breakdown and relative weights of specific cost items, nor did they provide associated resource use data (e.g., hours of trial staff time used), both of which would be required for insights into potential efficiency gains [24]. Those sort of insights have only been derived from simulation studies investigating strategies to reduce RCT costs [12, 25] and the intuition of experienced trial leaders [2, 14]; both of which have repeatedly criticized extensive on-site monitoring as a major cause for increasing costs of RCTs and suggested more streamlined approaches. Site monitoring with source data verification was estimated to account for 15–30% of clinical trial costs in industry sponsored trials [12, 23, 25, 26]. Respective estimates for monitoring costs from investigator-initiated RCTs are, except for a recent case report by our group [27], not available. In a systematic review of studies reporting empirical data on resource use and costs of RCTs up to November 2016 by our group [22], we identified 56 articles that provided some information on resource use or costs in RCTs, but no study comprehensively presenting detailed resource use and associated costs for the entire course of an RCT. A more recent study used activity-based costing methodology to assess the estimated total costs of nine randomized phase 3 clinical trials with new anticancer biological agents [28]. Nevertheless, the current empirical evidence on the topic lacks comprehensiveness, transparency, detail, and a standardized methodology to assess consumed resources and costs. As a consequence, the usefulness of the available data for clinical researchers, academic institutions, biomedical companies, regulators, or funding agencies remains extremely limited.

In collaboration with several stakeholders, we developed a comprehensive costing list, with which we collected detailed data from two investigator-initiated RCTs to exemplify how costs and resource use can be systematically assessed and presented [27]. The final list comprised of fixed costs and variable costs built upon a list of study activities (activity-based costing) [29–31]. In both trials, personnel costs during patient enrolment, treatment, and follow-up accounted for the majority of expenditures. Data monitoring costs in both investigator-initiated RCTs, including source data verification, accounted for less than 1% of total costs, in contrast with 10–30% for industry trials [12, 23, 26]. Generalizing beyond these two trials, however, requires a large systematic investigation on cost structures and cost drivers in RCTs. Therefore, we aim in the present protocol to empirically investigate detailed resource use and cost data from a large sample of investigator-initiated RCTs in three countries.

## Study objectives and hypotheses

The aim of this study is to generate a database of detailed empirical resource use and cost data from 100 investigator-initiated RCTs in Switzerland, Germany, and the UK, so that we can descriptively investigate total RCT costs and proportions of total costs for individual cost items to achieve the following specific objectives:

A) To investigate cost patterns on an absolute (actual costs in USD) and relative scale (% of total costs) including largest cost items for the whole RCT and for the planning, conduct, and finalization phases.
B) To explore RCT costs across different strata including medical fields, intervention types, multicenter/single center status, and countries.
C) To examine planned versus actual RCT costs and to explore factors that explain excess costs.
D) To explore hidden costs associated with conducting an RCT such as resource use and costs for writing the protocol, trial committees, ongoing communication, and managing serious adverse events (SAEs).

We have the following specific hypotheses:

(i) The expenses for participant recruitment and follow-up typically constitute the largest proportion of total RCT costs.
(ii) Data monitoring constitutes—on average—less than 15% of total RCT costs.
(iii) Delays in recruitment of trial participants explains most of the excess costs.
(iv) Cost structures (proportions) and fixed cost items (e.g., regulatory or ethics committee approval) are similar across medical fields and countries.

### Study design

This is an observational study for which we aim to retrospectively collect detailed empirical cost and resource use data from 100 investigator-initiated RCTs from three different countries (Switzerland *n* = 60, Germany *n* = 20, UK *n* = 20). This study was registered on Open Science Framework [32]. This study does not collect any patient- or health-related data and does not require ethical approval (confirmed by EKNZ Project ID: Req-2024–00581).

### Eligibility criteria and trial identification

RCTs will be included if they have been completed or are close to completion (statistical analysis ongoing or manuscript in preparation). Trials will be excluded if they are industry sponsored, dose-finding RCTs, RCTs primarily evaluating pharmacokinetics or physiology, include healthy volunteers, or RCTs that published results more than 10 years prior to the start of this investigation. To enhance comparability across countries, we will aim to include 10–15 RCTs each from cardiovascular, oncology, surgical, and miscellaneous fields. However, it was challenging to find principal investigators willing to share their cost data, as such we decided to expand our scope and include a small sample of pilot studies (*n* = 10) and RCTs initiated in Switzerland but conducted in low- and lower-middle-income countries (*n* = 10).

We will identify potentially eligible RCTs using two approaches: Firstly, we will use a convenience sample and contact Clinical Trial Units (CTUs), trial networks, and individual principal investigators known to the authors in all three countries and ask them about eligible investigator-initiated RCTs they could contribute. Most of our direct trialist connections are in Switzerland. In particular, we will approach the Swiss CTU network; the KKS Network, UK Registered CTUs Network; Network of Hubs for Trials Methodology Research (UK); UK Trial Managers’ Network, Trial Forge Initiative (UK); Koordinierungszentren für Klinische Studien Network (DE); Oncology network (DE); and the Clinical Trials Centre Cologne (DE).

Secondly, we will screen trial registries for completed RCTs and then invite principal investigators to participate in our study. These trial registries will include clinictrials.gov, the ISRCTN registry, the Health Research Authority (NHS) registry, Kofam (Swiss Clinical trial registry), and the Deutsches Register Klinischer Studien (DRKS; the German clinical trial registry).

For each RCT contributed to the project, we will offer the trial teams 200 euros as a compensation for their efforts.

### Data collection procedures

Protocols and publications will be collected for each of the RCTs and the cost data will be collected and stored in the electronic data capture system REDCap [33, 34]. Trial teams will enter their cost and resource data directly into an online data collection form. Data entry will be followed by a short interview to clarify unclear values and missing data. A cost report will be generated for each trial, which is then reviewed by the trial team. Funders of RCTs were not contacted, since previous research has shown that budgets are purposefully underestimated and we wanted to record the actual costs of RCTs [35].

For the collection of the resource use and cost data, we will use a previously developed and tested cost item list developed by Speich et al. that follows principles of activity-based costing [27, 36]. An overview of the collected variables can be found in Table 1. All variables and the online data collection form can be found in the supplementary material (Supplement Table 1 and Supplement Table 2). The online cost report form will be tested on two RCTs and changes will be made based on the feedback provided by investigators and study teams.

**Table 1:** Total trial costs

**Total Trial Costs ** • Trial Budget • Trial Funding • Total trial costs • Total trial costs adjusted for employer contributions • Total trial costs adjusted for employer contributions and 30% unaccounted time • Total trial costs adjusted for employer contributions, 30% unaccounted time and overhead		
**Planning**	**Conduct**	**Finalization**
**Fixed Costs** • Ethics Committee approval fees • Health authority fees • Other approval fees • Costs for Infrastructure and Equipment • Shipping costs • Insurance fees	**Fixed Costs** • Advertisment • Costs of Intervention • Placebo manufacturing • Additional procedures and material • Patient reimbursement • Specimen analysis • AE/SAE/SUSAR material • Travel costs • Audits and Inspections	**Fixed Costs** • Publication fees • Conference fees and costs
**Variable Costs (in days)** • Research protocol • Budget development • Grants and funding • Application EC, • Patient-related forms • Investigational brochure • Site-specific set-up Staff training • Acquiring study drug • Statistical analysis plan • Data management plan • Monitoring plan • Initial monitoring Visit • Biobank preparation • Communication funders and authorities • Communication involved sites • Communication other stakeholders • Communication patient representatives	**Variable Costs (in days or minutes)** • Screening • Informed consent and patient specific documents • Randomization • Baseline visit • Treatment: intervention • Treatment: control • Sample processing/analysis • Source data and data entry AEs, SAEs and SUSARs • Follow-up/Endline visit(s): • Ongoing communication • Upkeep of documentation • staff training /re-training • Amendments • Audits/ inspections • statistical services • Data management • Monitoring - Upkeep of biobank	**Variable Costs (in days)** • Data cleaning and database lock • Biospecimen storage/destruction • Statistical analyses • Final reporting • Manuscript • Other results dissemination

*Abbreviations*: *AE* adverse event, *SAE* serious adverse event, *SUSAR* serious unexpected serious adverse event, *EC* ethics committee

In short, we will be collecting two types of cost data: Firstly, the fixed costs associated with the trial, which are costs that are independent of time and human resources, such as ethical approval fees, insurance fees, travel costs, and material fees (Table 1). Secondly, we will be collecting variable costs, using a predefined list of study tasks, which are dependent on human resources, time expenditure of the team and salary, such as days needed to write the protocol, days for communication amongst stakeholders, minutes needed for patient screening, recruitment, and treatment (Table 1). Variable costs will be recorded in days or minutes for each study task, for each type of trial team member (principal investigator, site investigator, study nurse/coordinator, senior medical staff, junior medical staff, administrative staff, statistician, IT support and data management, monitor, laboratory staff, or others) and will then be multiplied by their gross yearly income (given by the study team) to calculate billable hours/days. The costs can be entered in five currencies: euro (€), Swiss franc (CHF), pound (£), and US dollar (USD).

From protocols and publications, we will extract the relevant RCT baseline characteristics. These will include medical field (indication), type of intervention and control group, use of placebo, number of arms, type of patient population, specific trial design (e.g., cross-over or factorial design), number of trial arms, number of trial sites, planned and achieved sample size, originally planned total costs and planned budget (if available), primary outcome(s), trial duration, planned and actual recruitment duration, monitoring method, electronic or paper-based data capture/case report forms, and funding sources.

### Sample size considerations

**Fig. 1:**
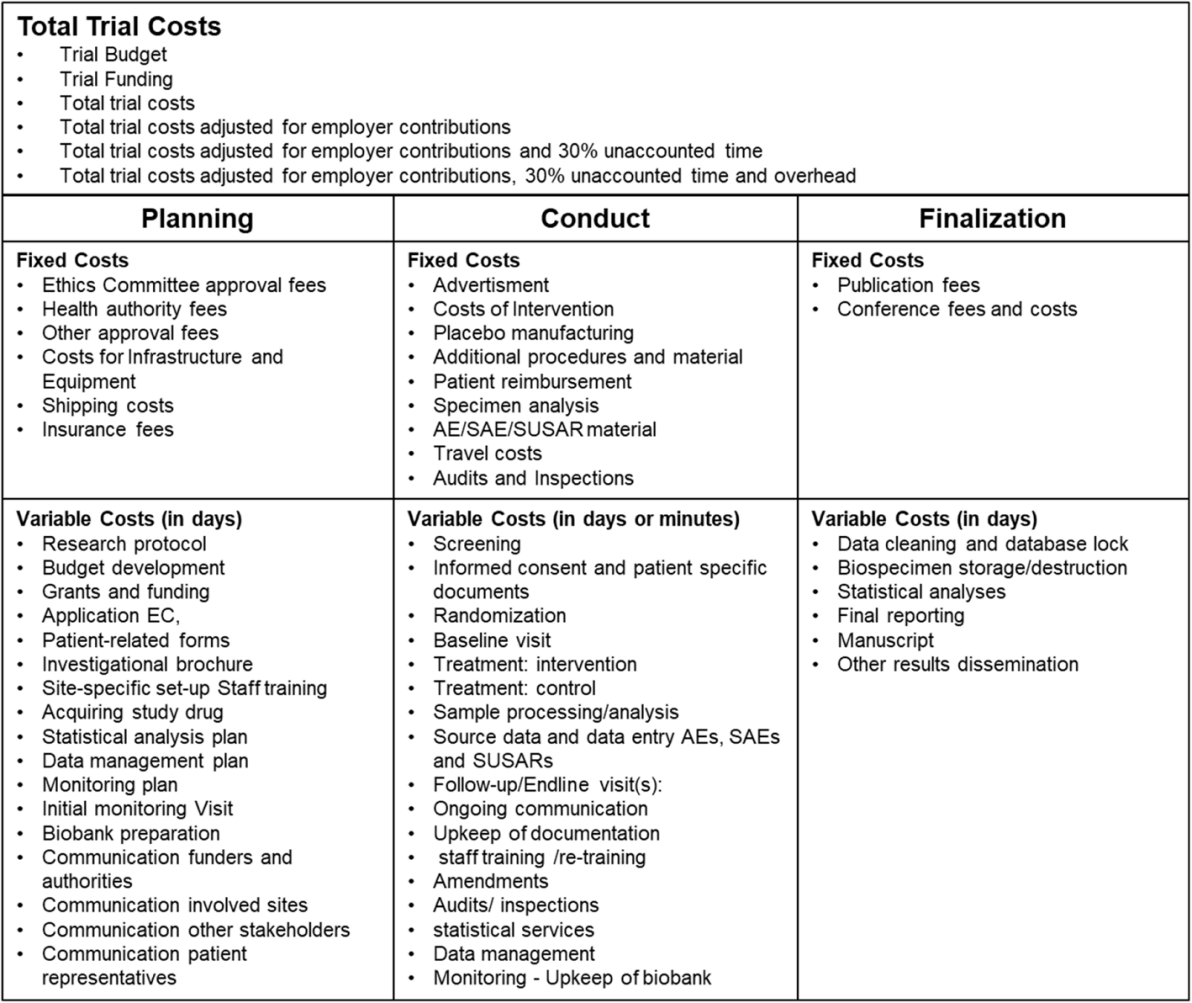
The required sample size (on the y-axis) required to achieve, with a probability of 90%, an estimate of the mean item cost proportion that has a confidence interval (CI95) smaller than ± 3%, as a function of the true mean item cost proportion (on the x-axis). The different lines represent different assumptions about the distribution of cost proportions across studies

We simulated data sets with various numbers of RCTs to study how many RCTs we would need to achieve a 90% probability (statistical power) for a sufficiently narrow 95% confidence interval (CI) around the estimated mean cost proportions for individual cost items. We assumed that a 95% CI width of 6% (i.e., a maximum of ± 3%) for the estimated mean would be sufficiently precise. The confidence interval assumes a normal distribution and is calculated as 1.96 * the standard error. Figure 1 shows how the required sample size depends on the true average proportion. Note that there are two lines: a dark line for a rather wide assumed distribution of proportions (tau = 10; beta distribution with a precision of 10) and a gray line for a plausible, narrower distribution (tau = 20). According to the dark line (the wider distribution), including a total of 60 RCTs would suffice to achieve a power of 90% to find a satisfyingly narrow 95% CI of 6% for a mean proportion below 0.15. Thus, our original plan was to include at least a total of 180 RCTs which appeared appropriate to achieve sufficient precision for estimated proportions (even within subgroups such as countries). However, it proved extremely challenging to find principal investigators willing to share detailed resource use and cost data of their trials rendering the target sample size of 180 RCTs unfeasible. Therefore, we reduced our target to 100 RCTs (Switzerland *n* = 60; Germany *n* = 20; UK *n* = 20). This number is feasible and will still allow for sufficient precision of unstratified estimates and to conduct multivariable regression analyses without overfitting models.

## Data management

All data will be collected for each of the RCTs and the cost data will be collected and stored in the electronic data capture system REDCap [33, 34]. For the statistical analysis, we will transfer data to the statistical software R version 3.5.1.

The variable costs will be adjusted for employer contributions (%), the overhead of the sponsor institution (%), and for unaccounted time (%). Employer contributions are additional expenses and contributions associated with employing a person, such as social security contributions, pension contributions, health insurance, and other benefits that are part of an employee’s compensation package in addition to their base salary; specifically, 23.3% of the gross salary in Germany [37], 15.0% in Switzerland [38], and 21.3% in the UK [39]. Unaccounted time is defined as the time spent on study activities not recorded or accounted for by the study team and was determined as 30%, based on results of a study shadowing clinical investigators during their study activities [40]. The overhead for the institution was recorded in the online data collection form by the study team. All costs will be converted into USD using historical exchange rates provided by the European Central Bank [41]. We will use the start date of recruitment as the date for the historical exchange rate and convert the costs to values for the year 2022. Values will also be adjusted for inflation to the year 2022 using the World Bank inflation index [42].

In Switzerland, a workday was defined as 9.6 h for medical staff (principal investigator, site investigator, medical staff junior, medical staff senior) and 8.4 h for other staff members (research coordinator, statistician, IT support and data management, monitor, laboratory staff), with a total of 226 workdays per year (average number of workdays in Switzerland). In Germany and the UK, a workday was defined as 9.6 h for medical staff and 7.5 h for other staff members, with a total of 223 workdays per year in Germany and 224 work days per year in the UK.

### Statistical analysis

We will calculate proportional contributions to total costs for individual cost items and for the three pre-defined study phases (preparation, conduct, and finalization). Proportions across RCTs will be summarized with descriptive statistics accompanied by 95% CIs. We will summarize total resource use (working hours and costs), as well as resource use and costs for individual cost items, and costs per patient using descriptive statistics (means or medians accompanied by standard deviations (SDs) or 25th to 75th percentile ranges [IQRs] according to the distribution, minimum [min] and maximum [max], respectively) overall and stratified by RCT over- or under-budget (± 10%), medical field, intervention type, country, and pilot vs non-pilot RCTs (objectives A–D).

We will categorize reasons for going over- or under-budget as reported by trial investigators and reasons for prolongation of recruitment [43] (objective C). In sensitivity analyses, we will calculate results without adding 30% of unaccounted time and/or adding overhead costs. We will not exclude any collected data from planned analyses. Outliers will be reviewed and checked/verified with principal investigators of included trials.

We will conduct two multivariable linear regression analyses with total costs in US dollars and total costs per patient as dependent variables [44]. Medical field, country, number of trial sites, actual sample size (only in regression model for total costs), routine data use, intervention type, and actual trial duration will be used as independent variables in both regression analyses. As for the investigation into the strata of cost proportions of RCTs across medical fields, intervention types, and countries, we will first plot distributions of cost proportions graphically stratified by medical field, intervention type, and country. For each RCT, we will descriptively compare the originally planned versus actual total costs, the originally planned versus actual recruitment duration, and the planned versus actual sample size (objective C).

We will calculate excess costs for each RCT (delta of originally planned total costs and actual total costs [Total costs − Planned Costs]). This will help investigate trial characteristics associated with excess costs of an RCT (dependent variable). We will develop a multivariable regression model with delta in recruitment duration, actual sample size, and actual number of trial sites, country, intervention type, and medical field as independent variables (objective C).

#### Missing data

Throughout the study we will be in direct contact will principal investigators and all data entry will be followed by a detailed cost report and interview. As such, we do not anticipate missing data. Nevertheless, some investigators may not have calculated an initial budget for their trials, i.e., in these cases we will document missing budgets and for analyses in which initial budgets are considered we will conduct complete case analyses. If income was not provided by the institution to calculate billable hours, we will impute the median income for the respective country, for each specific team role.

#### Changes to the original OSF registration

In our initial OSF registration, we had planned to include an additional sample of Canadian RCTs. Due to feasibility issues in Canada and lack of support from collaborators, we needed to exclude the country. Furthermore, it was challenging to find principal investigators willing to share their cost data, as such we decided to expand our scope and include a small sample of pilot studies (*n* = 10) and RCTs initiated in Switzerland but conducted in low- and lower-middle-income countries (*n* = 10). In addition, we initially did not want to include trials published later than 5 years; this was extended to 10 years to increase our sample of eligible RCTs.

### Confidentiality

We will inform principal investigators of included trials that only aggregated data will be published and none of the individual RCTs, investigators, staff members, or sponsors will be identifiable. A standard data sharing agreement was consented to by both parties. If required by the institution, a more detailed data sharing agreement was written and signed by both departments. The data was stored on a secure online database (REDCap) and stored on a safe University Hospital Basel server. Access to this database is limited and password protected. PIs only had access to their data entry form.

## Discussion

This empirical study will help guaranteeing the sustainability of investigator-initiated RCTs in the future. The Making Clinical Trials more Affordable (MARTA) working group has prepared for the present study with previous projects: An interview study published by McLennan et al. shed light on the systemic underfunding of investigator-initiated clinical trials in Switzerland, identifying inaccurate budget estimations and limited funding sources as key challenges [45]. The authors highlight the need for transparency on trial costs as well as training in budgeting practices [45]. Another study explored the use of electronic health records and their potential for cost reduction by supporting recruitment and outcome assessment in RCTs [46]. A scoping review has systematically identified several freely available tools for planning and monitoring of RCT costs, emphasizing the need for user-testing and validation of tools [47]. Additionally, a meta-research study investigated the challenges in obtaining placebos, highlighting the substantial resources required [48]. Together, these studies contribute to the ongoing conversation on optimizing the planning, funding, and execution of clinical trials for more affordable and effective research practices. Nevertheless, empirical data on RCT costs of investigator-initiated RCTs are still missing and urgently needed. The present effort will help identify areas for cost reduction, improve future funding of RCTs, and will help investigators plan their trials by providing real cost and time expenditure estimates.

As a major strength, this study will be the first of its kind to use activity-based costing to systematically and empirically investigate the resource use and costs of RCTs in Germany, Switzerland, and the UK. However, the study also has limitations. Firstly, despite including trials from different medical fields and countries, our findings may not be generalizable to all types of trials or research settings, as the costs can vary widely depending on the research question, therapeutic area, and location. Secondly, we will collect self-reported retrospective data from investigators and their teams, which may introduce reporting bias and inaccuracies. Especially the variable costs (time expenditures) are not usually recorded on site and need to be estimated by the principal investigator and study team members. Thirdly, RCTs of principal investigators who agreed to share the resource use and cost data with us might not be representative of all RCTs, but could be a selection of more successful RCTs compared with those for which principal investigators did not share cost data. We will keep a list of all contacted trial investigators, who do not wish to participate in our study, and will extract characteristics of corresponding not-included RCTs from trial registries and publications to enable a comparison of RCT characteristics. Fourth, our sample only includes trials conducted in Europe and costs may differ in North America or Asia because of differences in healthcare systems, medical research, and health insurance policies. Lastly, our research does not investigate if the different monitoring strategies have an influence on data quality.

Future research should consider alternative methodologies such as prospective data collection to provide more accurate cost estimates.

### Study status

We have currently (January 2021 to December 2023) collected resource use and cost data of 87 investigator-initiated RCTs (Switzerland *n* = 43; Germany *n* = 20; UK *n* = 16; and low- and lower-middle-income countries *n* = 8).

## Supplementary Information

Supplementary Material 1.

## Data Availability

For this protocol, all data is presented in the manuscript. Cost data of this project is confidential upon agreement with the principal investigators. As such, only aggregated data will be shared.
