# Supplementary material for: Design and rationale for an empirical investigation of the resource use and costs of investigator-initiated randomized trials in Switzerland, the UK, and Germany
Source: Trials. 2024 Oct 7;25:662. doi: 10.1186/s13063-024-08505-y (PMC11457363; doi:10.1186/s13063-024-08505-y)
Supplement: Supplementary file 1 — Supplementary Material 1. [file 13063_2024_8505_MOESM1_ESM.docx]

### Supplementary Material: Design and rationale for an empirical investigation of resource use and costs of investigator-initiated randomized controlled trials in Switzerland, the United Kingdom, and Germany

**Authors**

Alexandra Griessbach MSc^1^

Benjamin Speich PhD^1^

Gilles Dutilh PhD

Shaun Treweek PhD^2^

Matthias Schwenkglenks PhD MPH^3^

Matthias Briel MD PhD^1,4^

**Link to CRF:** [**https://redcap.link/RCT_Costs**](https://redcap.link/RCT_Costs)

**Table 1: Baseline Characteristics**

| **Instrument: Study Baseline** | | | **(study_baseline)** | | | | | | | | | | | |
| --- | --- | --- | --- | --- | --- | --- | --- | --- | --- | --- | --- | --- | --- | --- |
|  | 1013 | [d1] |  | Section Header: *1. Publication*  [title] | descriptive | | | | | | | | | |
|  | 1014 | [d2] |  | [reg_v2] | descriptive | | | | | | | | | |
|  | 1015 | [upload_results] |  | Upload Results publication | le | | | | | | | | | |
|  | 1016 | [status_other] |  | Section Header: *2. Study Information and Design*  Status of trial | radio | | | | | | | | | |
|  |  |  |  |  | 1 | Completed | | | | |  | | | |
|  |  |  |  |  | 2 | Discontinued | | | | |  |  |  |  |
|  |  |  |  |  | 3 | Other | | | | |  |  |  |  |
|  | 1017 | [pilot] |  | Is this a Pilot trial? | truefalse | | | | | | | | | |
|  |  |  |  |  | 1 | True | | |  | | | | | |
|  |  |  |  |  | 0 | False | | |  |  |  |  |  |  |
|  | 1018 | [discontinued_reason]  Show the eld ONLY if: [status_other] = '2' | | Reason: | checkbox | | | | | | | | | |
|  |  |  |  |  | 1 | | discontinued_reason___1 | | | | | | Di culty in recruitment | |
|  |  |  |  |  | 2 | | discontinued_reason___2 | | | | | | Financial di culties | |
|  |  |  |  |  | 3 | | discontinued_reason___3 | | | | | | New results leading to decision to stop trial | |
|  |  |  |  |  | 99 | | discontinued_reason___99 | | | | | | Other | |
|  | 1019 | [other_status]  Show the eld ONLY if: [status_other] = '3' | | specify other | notes | | | | | | | | | |
|  | 1020 | [list_funders] | | List of funders | notes | | | | | | | | | |
|  | 1021 | [phase] | | Phase of trial | checkbox | | | | | | | | | |
|  |  |  |  |  | 1 | phase___1 | | | | Early Phase | |  | | |
|  |  |  |  |  | 2 | phase___2 | | | | Late Phase | |  |  |  |
|  |  |  |  |  | 3 | phase___3 | | | | Not sure | |  |  |  |
|  | 1022 | [compar_effect] | | Comparative e ectiveness? | yesno | | | | | | | | | |
|  |  |  |  |  | 1 | Yes | |  | | | | | | |
|  |  |  |  |  | 0 | No | |  |  |  |  |  |  |  |
|  | 1023 | [intervention] | | Type of intervention | radio | | | | | | | | | |
|  |  |  |  |  | 1 | | Investigational Medicinal Product (IMP)/Drug | | | | | | |  |
|  |  |  |  |  | 2 | | Medical Device | | | | | | |  |
|  |  |  |  |  | 3 | | Surgical | | | | | | |  |
|  |  |  |  |  | 4 | | Vaccine | | | | | | |  |
|  |  |  |  |  | 5 | | Behavioral | | | | | | |  |
|  |  |  |  |  | 6 | | Physical therapy/exercise | | | | | | |  |
|  |  |  |  |  | 7 | | Diagnostic | | | | | | |  |
|  |  |  |  |  | 9 | | Dietary supplement | | | | | | |  |
|  |  |  |  |  | 99 | | Other | | | | | | |  |

|  | 1024 | [unit_rand] | Unit of randomisation | radio | | | | | | | |
| --- | --- | --- | --- | --- | --- | --- | --- | --- | --- | --- | --- |
|  |  |  |  | 1 | Patients | |  | | | | |
|  |  |  |  | 2 | Clusters | |  |  |  |  |  |
|  |  |  |  | 3 | Body parts | |  |  |  |  |  |
|  |  |  |  | 4 | Physicians | |  |  |  |  |  |
|  | 1025 | [other_intervention]  Show the eld ONLY if:  [intervention] = '99' | specify other typ of intervention | text | | | | | | | |
|  | 1026 | [thera_area] | Therapeutic Area | dropdown | | | | | | | |
|  |  |  |  | 1 | | anaesthetics | | | |  | |
|  |  |  |  | 2 | | cardiothpracic surgery | | | |  |  |
|  |  |  |  | 3 | | cardiovascular | | | |  |  |
|  |  |  |  | 4 | | dermatology | | | |  |  |
|  |  |  |  | 5 | | ear nose throat(ENT) | | | |  |  |
|  |  |  |  | 6 | | endocirnology | | | |  |  |
|  |  |  |  | 7 | | gastointetinal | | | |  |  |
|  |  |  |  | 8 | | general surgery | | | |  |  |
|  |  |  |  | 9 | | hematology | | | |  |  |
|  |  |  |  | 10 | | infectious disease | | | |  |  |
|  |  |  |  | 11 | | neuphrology | | | |  |  |
|  |  |  |  | 12 | | neuro rehabiliation | | | |  |  |
|  |  |  |  | 13 | | neuro surgery | | | |  |  |
|  |  |  |  | 14 | | neurology | | | |  |  |
|  |  |  |  | 15 | | obstetrics/gynecology | | | |  |  |
|  |  |  |  | 16 | | oncology | | | |  |  |
|  |  |  |  | 17 | | opthalomogy | | | |  |  |
|  |  |  |  | 18 | | orthopedics | | | |  |  |
|  |  |  |  | 19 | | plastic surgery | | | |  |  |
|  |  |  |  | 20 | | pneumology | | | |  |  |
|  |  |  |  | 21 | | psychiatry | | | |  |  |
|  |  |  |  | 22 | | respiratory | | | |  |  |
|  |  |  |  | 23 | | rheumatology | | | |  |  |
|  |  |  |  | 24 | | urology | | | |  |  |
|  |  |  |  | 99 | | other | | | |  |  |
|  | 1027 | [thera_other_v2]  Show the eld ONLY if: [thera_area] = '99' | if other, please name therapeutic eld | text | | | | | | | |
|  | 1028 | [design] | Type of design | checkbox | | | | | | | |
|  |  |  |  | 1 | | design___1 | | Parallel | | |  |
|  |  |  |  | 2 | | design___2 | | Cross-Over | | |  |
|  |  |  |  | 3 | | design___3 | | Factorial | | |  |
|  |  |  |  | 4 | | design___4 | | Cluster | | |  |
|  |  |  |  | 5 | | design___5 | | Adaptive | | |  |
|  |  |  |  | 99 | | design___99 | | Other | | |  |
|  | 1029 | [other_design]  Show the eld ONLY if: [design(99)] = '1' | specify other type of design | text | | | | | | | |
|  | 1030 | [type_ana] | Type of analysis | radio | | | | | | | |
|  |  |  |  | 1 | | superiority | | |  | | |
|  |  |  |  | 2 | | non-inferiority | | |  |  |  |
|  |  |  |  | 99 | | Other | | |  |  |  |
|  | 1031 | [other_analysis]  Show the eld ONLY if: [type_ana] = '99' | specify other type of analysis | text | | | | | | | |

|  | 1032 | [international] | National or international | radio | | | | | | | |
| --- | --- | --- | --- | --- | --- | --- | --- | --- | --- | --- | --- |
|  |  |  |  | 1 | national | | | | |  | |
|  |  |  |  | 2 | international | | | | |  |  |
|  | 1033 | [n_centers_2]  Show the eld ONLY if: [international] = '2' | List of countries | text | | | | | | | |
|  | 1034 | [multi] | Single or multi-center trial? | radio | | | | | | | |
|  |  |  |  | 1 | single | | |  | | | |
|  |  |  |  | 2 | multi | | |  |  |  |  |
|  | 1035 | [n_centers]  Show the eld ONLY if:  [multi] = '2' | How many centers? | text (number) | | | | | | | |
|  | 1036 | [blind] | Blinded | checkbox | | | | | | | |
|  |  |  |  | 0 | | blind___0 | | | None | |  |
|  |  |  |  | 1 | | blind___1 | | | Patient blinded | |  |
|  |  |  |  | 2 | | blind___2 | | | Care giver blinded | |  |
|  |  |  |  | 3 | | blind___3 | | | Outcome assessor blinded | |  |
|  |  |  |  | 99 | | blind___99 | | | Other | |  |
|  | 1037 | [other_blind]  Show the eld ONLY if:  [blind(99)] = '1' | specify other blinding | text | | | | | | | |
|  | 1038 | [interim] | Interim Analyses | yesno | | | | | | | |
|  |  |  |  | 1 | Yes | |  | | | | |
|  |  |  |  | 0 | No | |  |  |  |  |  |
|  | 1039 | [num_interim]  Show the eld ONLY if: [interim] = '1' | If yes, how many? | text (number) | | | | | | | |
|  | 1040 | [explor] | Substantial costs or time expenditure for laboratory , biospecimen, exploratory or genetic outcomes? | yesno | | | | | | | |
|  |  |  |  | 1 | Yes | |  | | | | |
|  |  |  |  | 0 | No | |  |  |  |  |  |
|  | 1041 | [population] | Section Header: *3. PICO*  Description Population | notes | | | | | | | |
|  | 1042 | [num_arms] | Number of arms (including control) | text (number) | | | | | | | |
|  | 1043 | [placebo_yn] | placebo | yesno | | | | | | | |
|  |  |  |  | 1 | Yes | |  | | | | |
|  |  |  |  | 0 | No | |  |  |  |  |  |
|  | 1044 | [control] | Description Control | notes | | | | | | | |
|  | 1045 | [desc_int]  Show the eld ONLY if: [num_arms] >= 2 | Description intervention 1 | notes | | | | | | | |
|  | 1046 | [desc_int_2]  Show the eld ONLY if: [num_arms] >= 3 | Description intervention 2 | notes | | | | | | | |
|  | 1047 | [desc_int_3]  Show the eld ONLY if: [num_arms] >= 4 | Description intervention 3 | notes | | | | | | | |
|  | 1048 | [desc_int_4]  Show the eld ONLY if: [num_arms] >= 5 | Description intervention 4 | notes | | | | | | | |
|  | 1049 | [desc_int_5]  Show the eld ONLY if: [num_arms] >= 6 | Description intervention 5 | notes | | | | | | | |
|  | 1050 | [desc_int_6]  Show the eld ONLY if: [num_arms] >= 7 | Description intervention 6 | notes | | | | | | | |
|  | 1051 | [outcomes] | Description Primary Outcome(s) | notes | | | | | | | |

**Table 2: CRF online Survey**


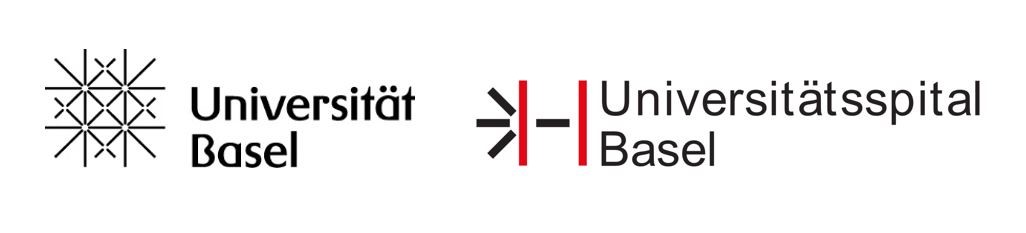


**Page 2 of 2**

**2**

**. Study Information**

**Please verify and complete data**

**O**

**cial Study Title**

* must provide value

**Study Acronym**

(

if applicable

)

**Registration Number**

e.g. Study registration number: clinicaltrial.gov, EUDRA CT, ISRCTN

**Country of main Investigational Site**

**Source of Funding**

Public

Industry

Both

**In what c**

**will you be lling in the data?**

**urrency**

€ (Euro)

C$ (Canadian Dollar)

£ (Pound)

CHF (Swiss Franc)

$ (US Dollar)

**Please upload your Study Protocol**

(

We will extract important trial charachteristics from the

protocol and publication of your trial)

**Were Results published for this trial?**

Yes

No

Planned

**Comments**

**Page 3/9**

A

A

A





<<

Previous Page

Submit

Save & Return Later

Powered by REDCa

[p](https://projectredcap.org/)

|  | 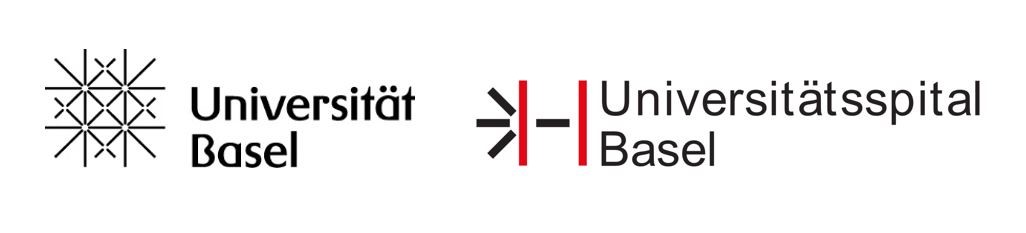  **Do not forget to save/submit at the end of the page before leaving.**  **Hint:** If you wish to return to a previous section of the survey, you can navigate to di erent parts by clicking the top right corner. |  **Survey Queue** |  |
| --- | --- | --- | --- |
|  |  | A A A     **"survey queue"** in |  |
|  | **3. Planned Budget and Fixed Costs vs. Variable Costs**  Planned Budget and Funding  **What was the planned budget for this trial?**  (you may also enter a range or if no budget was calculated enter**______**^£^  "unknown")  **How much funding did you receive for this trial? ________**£  (can be the same amount as initially budgeted)  **Was the design of the trial adapted to a pre-determined budget?**  Yes  Yes  No  **Was there non-monetary support?**  (provision of study drug or laboratory tests by industry or NGO) No | |  |
|  | Fixed Costs vs. Variable Costs (time expenditure) | |  |
|  | **1. Did you pay a xed amount for statistical services or was it recorded by the hour (time expenditure)?**  (statistical analysis plan, sample size calculation, interim analyses, nal analyses, graphics, etc.)  * must provide value  Recorded in hours Fixed amount paid Both | |  |
|  | **2. Did you pay a xed amount for monitoring or was it recorded by the hour (time expenditure)?**  (monitoring plan, all monitoring visits, drug accountability, data verication, travel, etc.)  * must provide value  Recorded in hours Fixed amount paid Both | |  |
|  | **3. Did you pay a xed amount for data management or was it recorded by the hour (time expenditure)?**  (Data management plan, development of CRF, eCRF, data cleaning, export, review, archiving, etc.)  * must provide value  Recorded in hours Fixed amount paid Both | |  |
|  | **4. Did you use biobanking services?**  (biobanking plan preparation, sample processing, storage, archiving, destruction, etc.)  * must provide value  Yes, recorded in hours Yes, xed amount paid Both  No | |  |


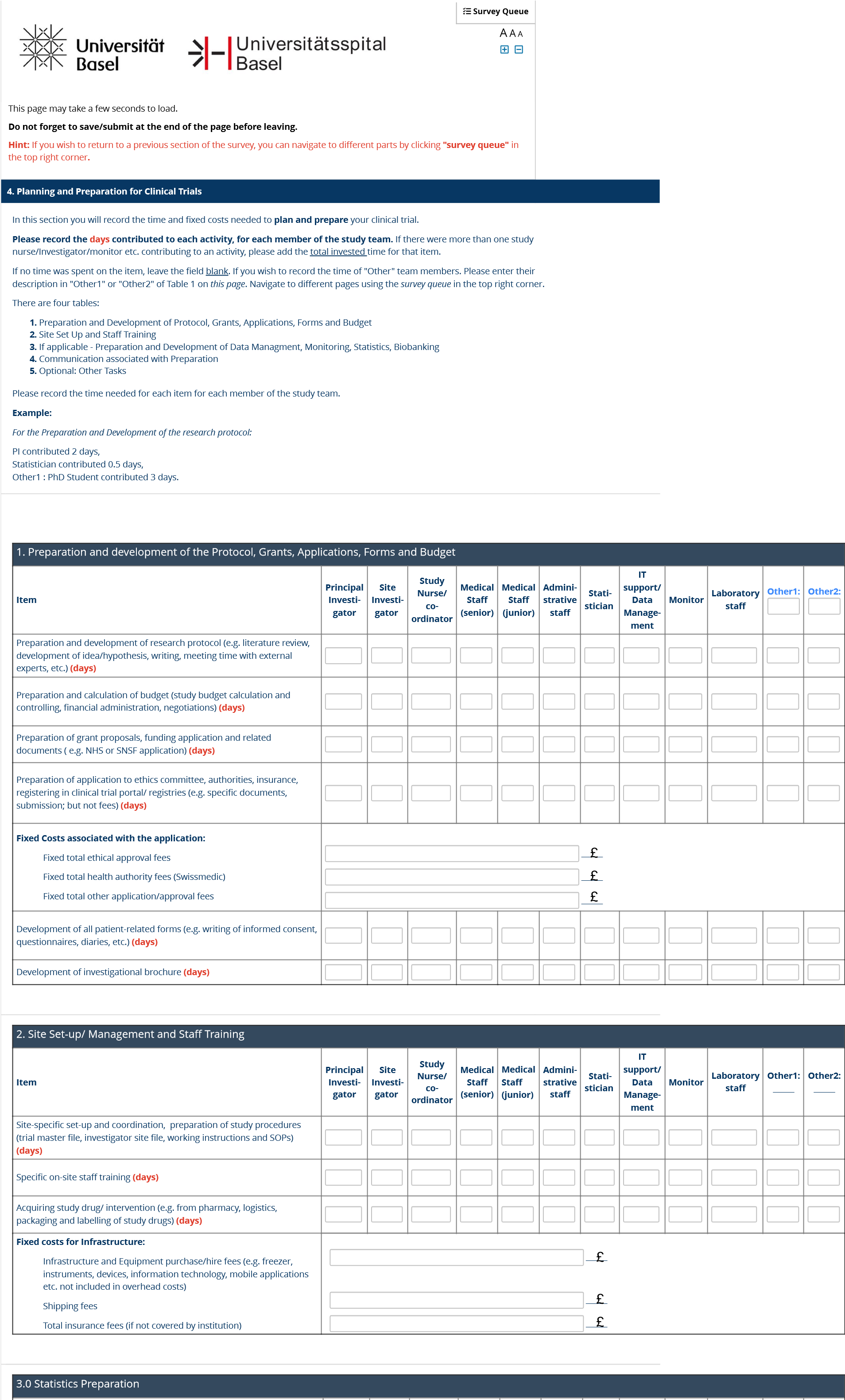


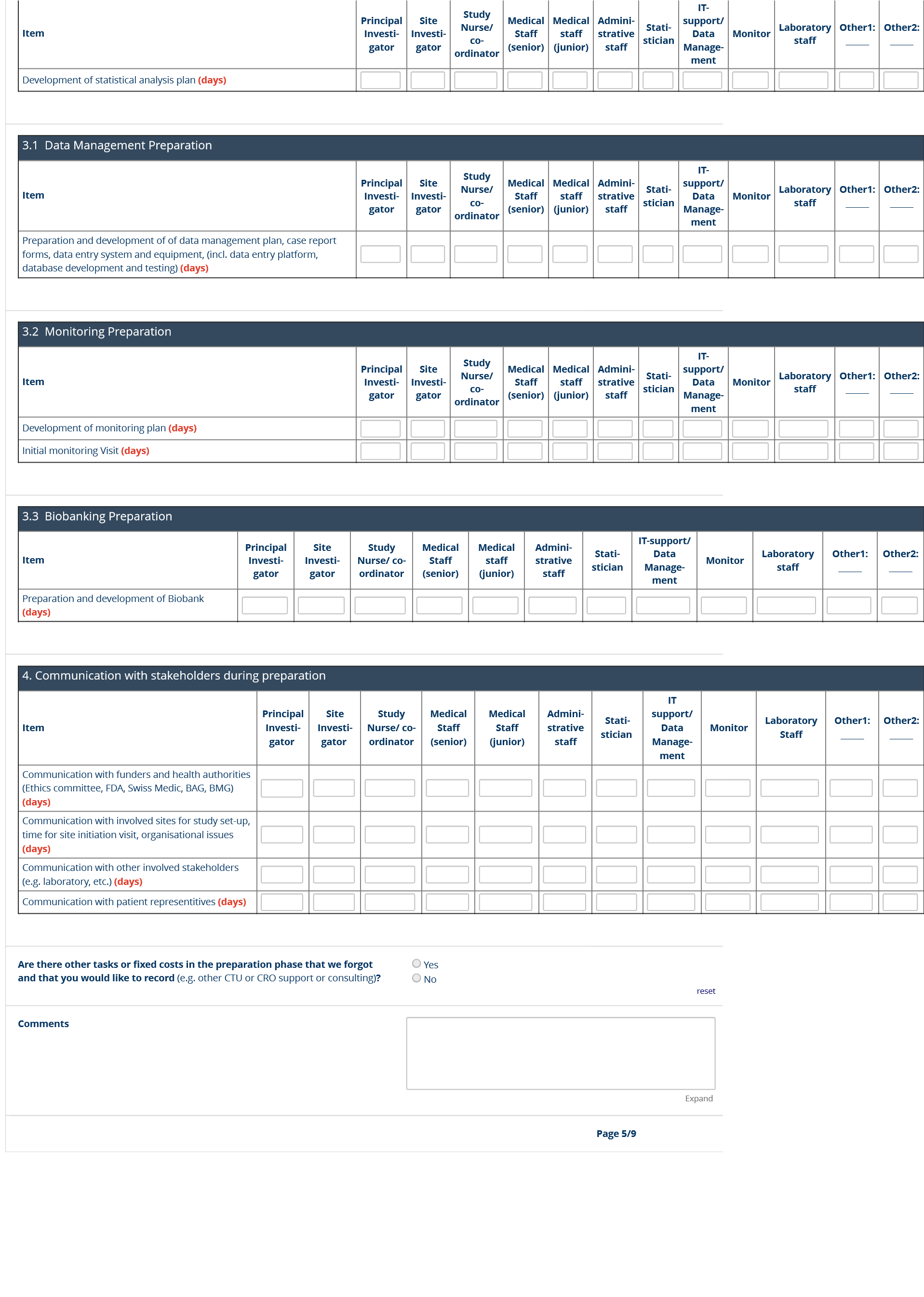


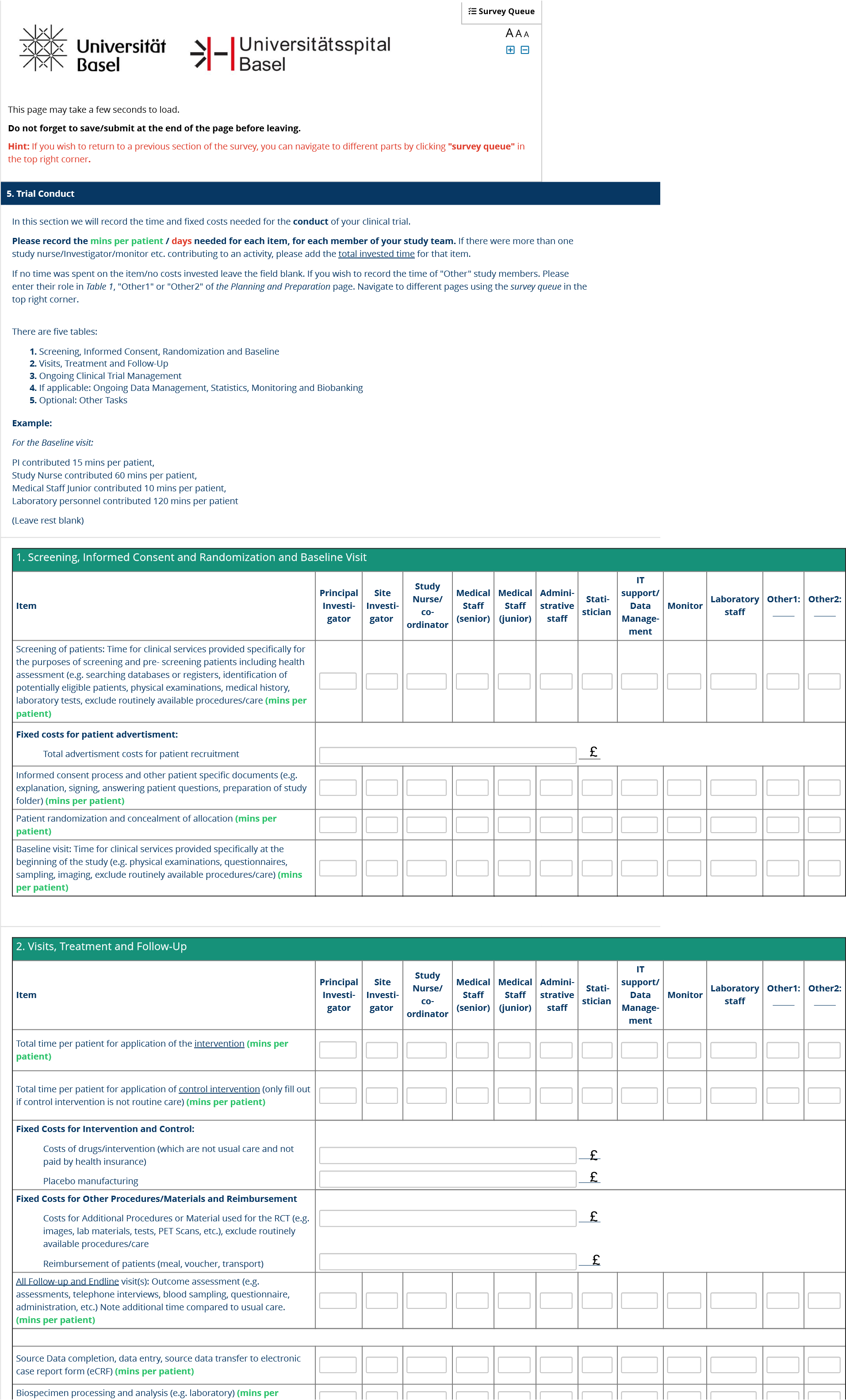


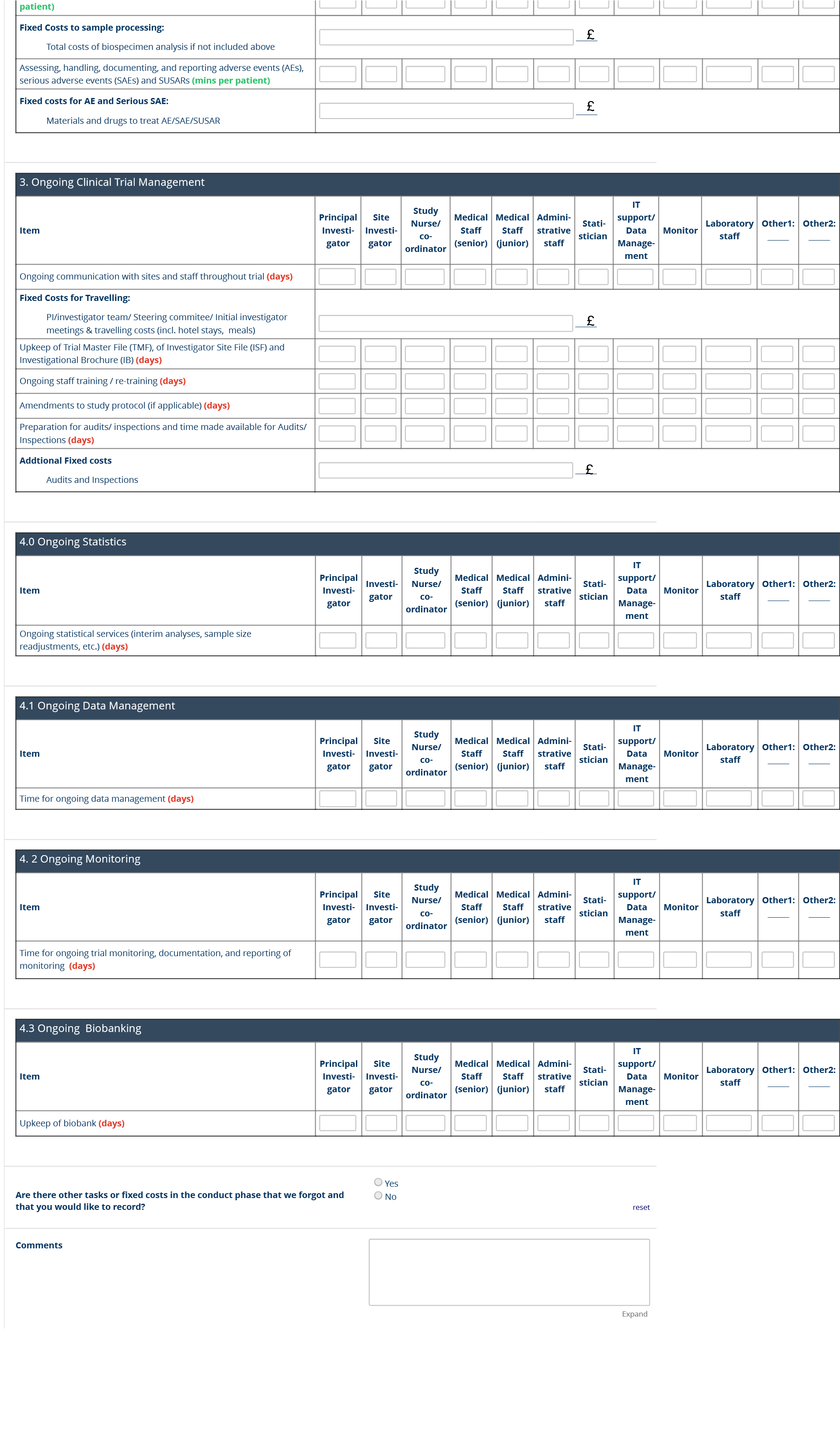


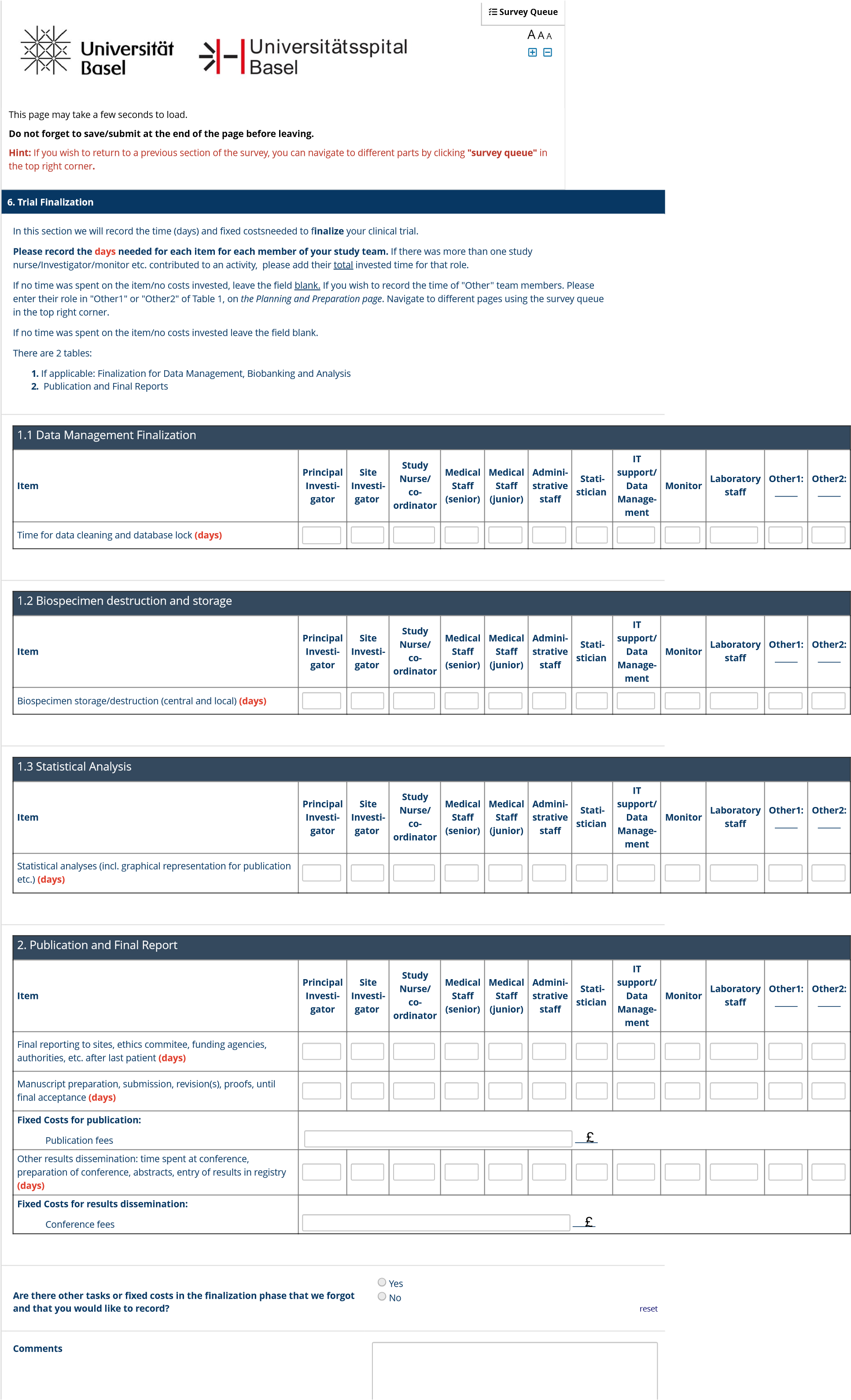


| 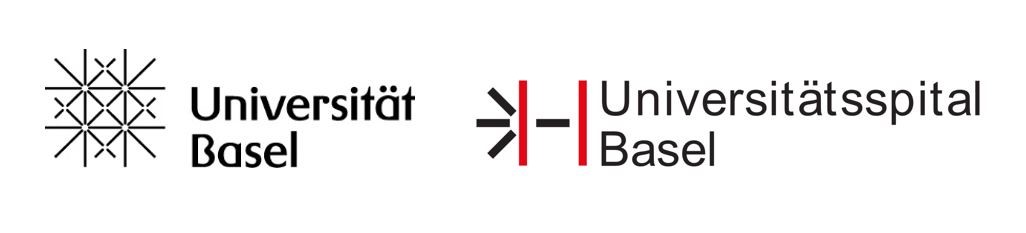  **Don't forget to save/submit at the end of the page before leaving.**  **Hint:** If you wish to return to a previous section of the survey, you can navigate to di erent parts by clicking **"survey queue"** in the top right corner**.** |  **Survey Queue** |
| --- | --- |
|  | A A A    |
| \| **7. Committees** \| \| --- \| | |
| **Did you use the services of one of these committees?** (check all that apply)  None  Trial Management/Steering Committee  Endpoint Assessment/Adjudication Committee  Data and Safety Monitoring Committee  Other | |
| \| **Committee** \| **Number of members in committee** \| **Total number of hours invested by members** \| **Were members reimbursed?** \| **If yes, how much?** (total for all members) \| \| --- \| --- \| --- \| --- \| --- \| \| **Trial Management/**  **Steering Committee** \|  \| **(hours)** \| Yes  No \| **______** \| \| **Endpoint Assessment/**  **Adjudication**  **Committee** \|  \| **(hours)** \| Yes  No \| **______** \| \| **Data and Safety**  **Monitoring Committee** \|  \| **(hours)** \| Yes  No \| **______** \| \| **Specify Other:** \|  \| **(hours)** \| Yes  No \| **______** \| | |
| \| **8. Sta Salaries and Overhead Costs** \| \| --- \| | |
| **What are the typical overhead costs for your institution? (in %, added to sta costs) %**  (if there is more than one overhead cost for dierent institutions, please enter the *mean* here and further list the overhead costs per institution in the comment box at the end of the page)  **Please enter the salary of the sta member(s) at the time of the trial**   \| **Sta** \| **Yearly gross (brutto) salary in ______** \| \| --- \| --- \| \| **Coordinating PI** \|  \| \| **Site investigator** \|  \| \| **Research nurse / coordinator** \|  \| \| **Medical sta (senior)** \|  \| \| **Medical sta (junior)** \|  \| \| **Administrative sta** \|  \| \| **Statistician** \|  \| \| **IT support / Data manager** \|  \| \| **Monitor** \|  \| \| **Laboratory sta** \|  \| \| **Other 1: ______** \|  \| \| **Other 2: ______** \|  \| | |
| **Comments** | |
| **Page 8/9** | |
|  | |

| 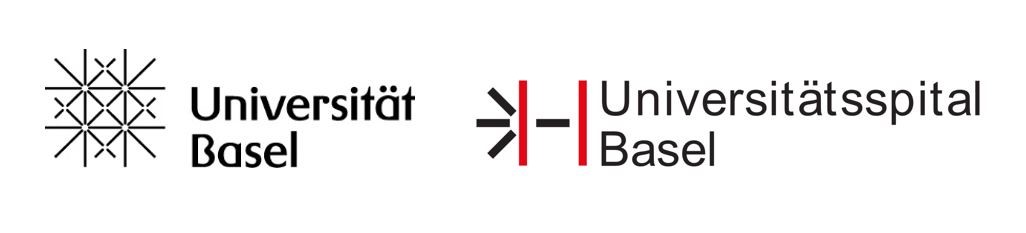  **Don't forget to save/submit at the end of the page before leaving.**  **Hint:** If you wish to return to a previous section of the survey, you can navigate to di erent parts by clicking **"survey queue"** in the top right corner**.** |  **Survey Queue** |
| --- | --- |
|  | A A A    |
| **Please verify and complete data** | |
| \| **9. Planned Sample Size, Screening, Recruitment and Trial Duration** \| \| --- \| | |
| \| Planned Sample Size \| \| --- \|   **Number of patients needed according to sample size calculation** | |
| \| Screening \| \| --- \|   **Number of patients screened** | |
| \| Randomization, Treatment and Lost to Follow-Up \| \| --- \|   **Number of patients randomized**  **Number of patients treated in intervention group(s)**  **Number of patients treated in control group(s)**  **Number of study visits** (per patient)  **Number of patients lost to follow-up** | |
| \| Trial Duration \| \| --- \|   **Planned trial start date** (based on registry)D-M-Y  **Planned trial end date** (based on registry)D-M-Y  **Recruitment start date** D-M-Y  **Recruitment end date**D-M-Y  **Date of last patient last visit** (LPLV)D-M-Y  **Date of publication** (epub date)D-M-Y | |
| \| **10. Other Study Information** \| \| --- \| | |
| **1. Did the recruitment of patients take longer or** Recruitment went as planned  **shorter than planned?** Longer  Shorter | |
| **2. Were the costs of the trial higher or lower than** Costs were as planned **your initially planned costs?** Higher than planned  Lower than planned  Were the costs higher than you initially planned? | |
| **3. Was a pilot or feasability study conducted?**  Yes No This is the pilot study | |
| **4. How many substantial amendments were necessary for this trial?** | |
| **5. How many Serious Adverse Events (SAE) and Suspected Unexpected Serious Adverse Reactions (SUSAR) occurred during the trial?**  Serious Adverse Events (SAE)  Suspected Unexpected Serious Adverse Reactions (SUSAR) | |
| **6a. Was the monitoring strategy determined by**  Yes No **the available trial budget?** | |
| **6b. What type of monitoring was used for the** none/ internal monitoring  **trial?** targeted with centralized monitoring  risk based monitoring with partial  source data veri cation  100% source data veri cation  Other | |
| **7. Was an electronic health record (EHR) or** Yes  **routinely collected data used?** No | |
| **8. What type of Case Report Form (CRF) was used?** electronic CRF (eCRF) paper CRF | |
|  | |
